# Europium Clustering and Glassy Magnetic Behavior in Inorganic Clathrate-VIII Eu_8_Ga_16_Ge_30_

**DOI:** 10.3390/ma15103439

**Published:** 2022-05-10

**Authors:** Nicolás Pérez, Manaswini Sahoo, Gabi Schierning, Kornelius Nielsch, George S. Nolas

**Affiliations:** 1 Institute for Metallic Materials, IFW-Dresden, 01069 Dresden, Germany; n.perez.rodriguez@ifw-dresden.de (N.P.); gschierning@physik.uni-bielefeld.de (G.S.); k.nielsch@ifw-dresden.de (K.N.); 2 Institute for Solid State, IFW-Dresden, 01069 Dresden, Germany; m.sahoo@ifw-dresden.de; 3 Department of Physics, Experimental Physics, Bielefeld University, 33615 Bielefeld, Germany; 4 Department of Physics, University of South Florida, Tampa, FL 33620, USA

**Keywords:** clathrate, thermoelectric, magnetic glass, transport

## Abstract

The temperature- and field-dependent, electrical and thermal properties of inorganic clathrate-VIII Eu_8_Ga_16_Ge_30_ were investigated. The type VIII clathrates were obtained from the melt of elements as reported previously. Specifically, the electrical resistivity data show hysteretic magnetoresistance at low temperatures, and the Seebeck coefficient and Hall data indicate magnetic interactions that affect the electronic structure in this material. Heat capacity and thermal conductivity data corroborate these findings and reveal the complex behavior due to Eu^2+^ magnetic ordering and clustering from approximately 13 to 4 K. Moreover, the low-frequency dynamic response indicates Eu_8_Ga_16_Ge_30_ to be a glassy magnetic system. In addition to advancing our fundamental understanding of the physical properties of this material, our results can be used to further the research for potential applications of interest in the fields of magnetocalorics or thermoelectrics.

## 1. Introduction

The common structural feature of all inorganic materials with a clathrate crystal structure is an open-structured, three-dimensional “host” lattice, or framework, that can encage “guest” atoms. The physical properties are directly related to the crystal structure, as well as the constituent elements. Furthermore, the effect of the guest species within the framework continues to be of interest as it plays an important role in the specific properties these materials possess, including low thermal conductivity, κ [1], superconductivity in *sp*^3^ bonded solids [2], magnetism [3,4,5,6], soft-modes [7,8], and tunneling [9,10]. The guest–host interactions constitute one of the most conspicuous aspects of these materials. Moreover, specific compositions continue to be investigated for certain applications of interest, such as optoelectronics [11,12] and potentially ultrahard materials [13]. Inorganic clathrates form in several different structural types [14], with clathrate-I compositions being studied most extensively, primarily due to their continuing interest for thermoelectrics applications [15,16,17].

Lanthanides constitute one of the guest constituents in inorganic clathrates, with europium-containing germanium clathrates revealing novel properties [9,10,18,19,20,21] and innovative applications in magnetic refrigeration, where magnetic ordering mediated by conduction electron spins has been reported [22]. The composition Eu_8_Ga_16_Ge_30_ can form in two completely different clathrate structure types, clathrate-I ($\mathrm{Pm}\bar{3}n$*)* and clathrate-VIII ($I4\bar{3}m$); the latter can be thought of as being formed by eight distorted pentagonal dodecahedra with vertices surrounding Eu^2+^ ions [3,5,14]. Herein we report on the temperature- and field-dependent electrical and thermal properties of clathrate-VIII Eu_8_Ga_16_Ge_30_. AC susceptibility, resistivity, Seebeck coefficient, Hall coefficient, thermal conductivity, and isobaric heat capacity measurements allowed us to describe the effects on the electronic structure with field, as well as the lattice dynamics of this material.

## 2. Materials and Methods

The synthesis of phase-pure, polycrystalline Eu_8_Ga_16_Ge_30_ has been reported previously [5]. Specimens were cut to appropriate sizes for different measurements, parallelepipeds of 1.8 × 1.8 × 8 mm^3^ for transport measurements and 1.8 × 1.8 × 0.5 mm^3^ for heat capacity measurements. The electrical and thermal transport measurements were performed at different applied magnetic fields in a commercial Quantum Design physical properties measurement system. These measurements have relative uncertainties of below 1% and 3% for the electrical and thermal measurements, respectively. Systematic deviations in the herein-reported values may increase up to 10% as a result of the combined uncertainties in determining lengths and weights. For resistivity and Hall measurements, thin indium wire (0.15 mm in diameter) was pressed onto the surface of the specimens. For κ measurements, thermally cured, Ag-loaded epoxy was used. The curing was accomplished at 170 °C in an Ar-atmosphere glovebox. For heat capacity measurements, the specimen was held in place on the calorimeter using a small amount of Apiezon N grease. For AC magnetic susceptibility measurements, an 11.6(7) mg specimen was zero-field cooled to 1.8 K in a Quantum Design magnetic property measurement system with data acquired upon heating at a rate of 0.3 K/min. Upon stabilizing the temperature, data were acquired with zero polarizing field (DC field) and a longitudinal excitation field (AC field) of 10 Oe at frequencies from 1 to 215 Hz.

## 3. Results and Discussion

**Figure 1:**
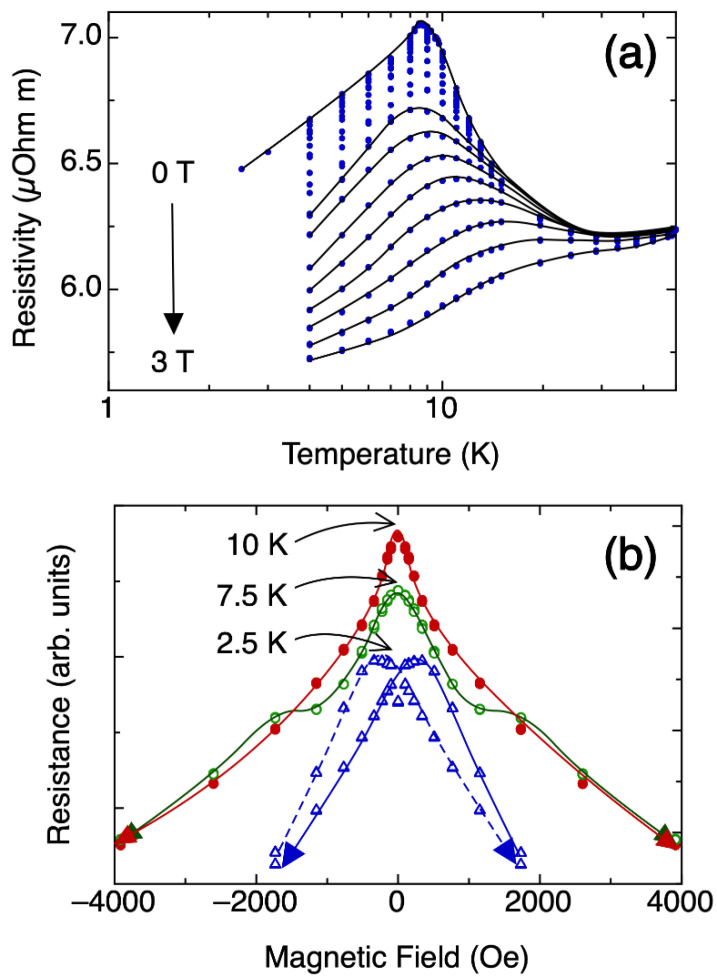
(**a**) Resistivity as a function of temperature in applied magnetic fields from 0 T to 3 T. The solid lines are a guide for the eye. (**b**) Resistance as a function of applied magnetic field for selected temperatures, with arrows indicating the direction of the change in the magnetic field.

Figure 1 shows the resistivity, ρ, data as a function of temperature and magnetic field. The peak associated with the ferromagnetic phase transition is readily observed at about 8.5 K [3]; however, noticeable magnetoresistance is observed above 30 K. For temperatures higher than 10 K, the magnetoresistance is anhysteretic, with a maximum at zero field that becomes more pronounced when approaching 10 K (Figure 1b). Below this temperature, an additional feature, characterized by a flattening of the curve, appears at +/− 2000 Oe. At 3 K, a clear hysteresis is observed, with a coercive field of approximately 500 Oe.

**Figure 2:**
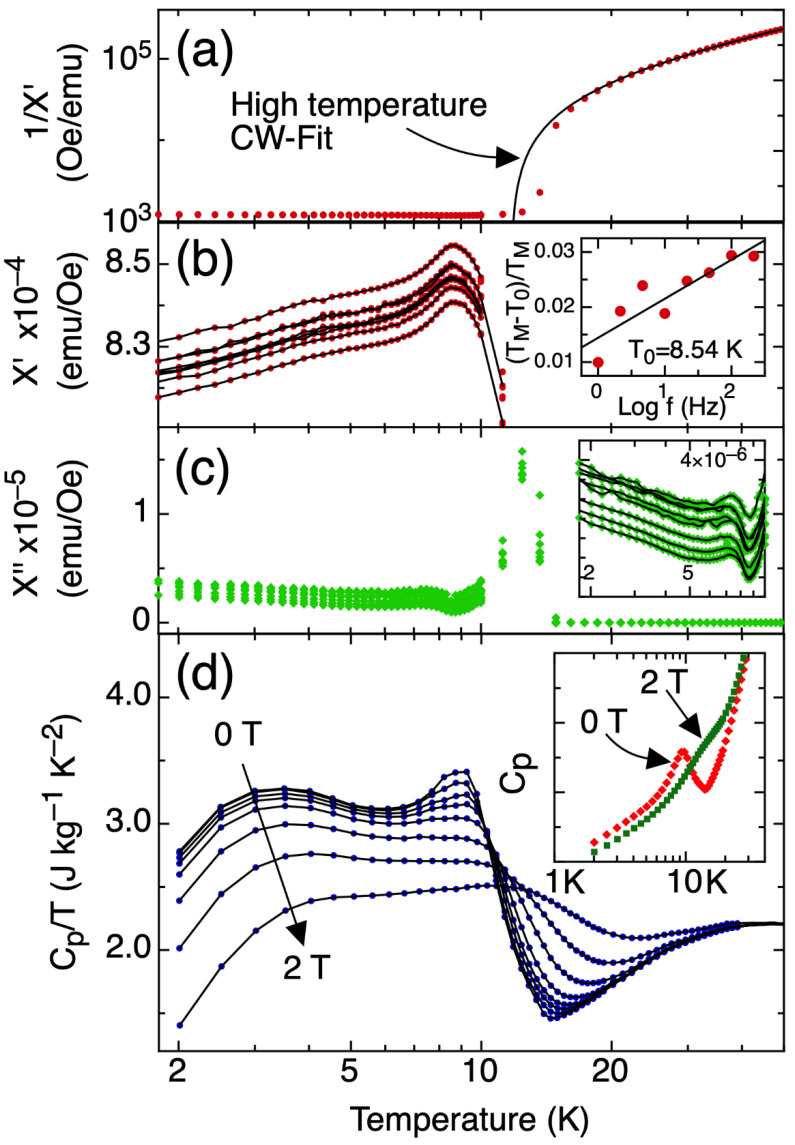
(**a**) The real part of the magnetic susceptibility with a solid line indicating the Curie–Weiss fit to the higher temperature data. (**b**) The real part of the AC magnetic susceptibility at low temperature. Inset: displacement of the peak with frequency. (**c**) The imaginary part of the AC magnetic susceptibility showing a peak at 13 K. Inset: detail of the data below 10 K. (**d**) C_p_/T as a function of temperature, with C_p_ vs. T in the inset, for applied magnetic fields of up to 2 T.

The real part of the AC susceptibility (Figure 2a) follows the Curie–Weiss (CW) Law at higher temperatures, reaching a plateau at 13 K, indicating the transition to ferromagnetic ordering. Deviation from the CW behavior is noticeable at temperatures higher than 20 K. Figure 2b shows the real part of the susceptibility in the ferromagnetic region with two features at 9 K and 4 K. The peaks in the imaginary part of the AC susceptibility (Figure 2c) indicate energy dissipation processes typically arising during magnetic order–disorder processes. As shown in Figure 2c, a clear peak is seen at about 13 K. In a previous study, a peak in magnetic entropy was reported for clathrate-VIII Eu_8_Ga_16_Ge_30_ at 13 K, with a second feature observed at about 9 K, and a shallow third feature at about 4 K [18]. Moreover, the feature at 9 K that corresponds to the reported transition temperature, which was also reported in resistance measurements from another study [3], shows evidence of slow dynamics since the peak shifts with frequency. The relative temperature shift per frequency decade of this peak was 7(1) × 10^−3^ (Figure 2b inset). A peak shift with temperature is one indication for magnetic clustering phenomena [23], and the low value obtained is compatible with a Ruderman–Kittel–Kasuya–Yosida (RKKY) interaction-mediated, glassy magnetic system [24]. This result is further supported by our magnetoresistance data (Figure 1), where the absence of hysteresis indicates the absence of long-range ferromagnetic ordering, the latter occurring below about 4 K [25].

Isobaric heat capacity, C_p_, as a function of temperature and magnetic field is shown in Figure 2d and indicates clear variation with applied magnetic field. In addition, two distinct regions are observed, corresponding to above and below the zero field maximum at 9 K. The representation of C_p_/T versus temperature allows one to visualize the evolution of entropy, indicating the relevant features of this phase transition more clearly. Between this maximum at 9 K and 40 K, C_p_/T increases with increasing applied magnetic field. At temperatures below the peak, C_p_/T decreases significantly with increasing applied magnetic field. This evolution is clear from the inset in Figure 2d, which compares C_p_ data for 0 T and 2 T. The two regions correlate well with the features in our ρ data (Figure 1). Moreover, an increase in the magnetic field has the apparent effect of displacing the position of the maximum peak towards higher temperatures in a similar manner as that shown in our ρ.

**Figure 3:**
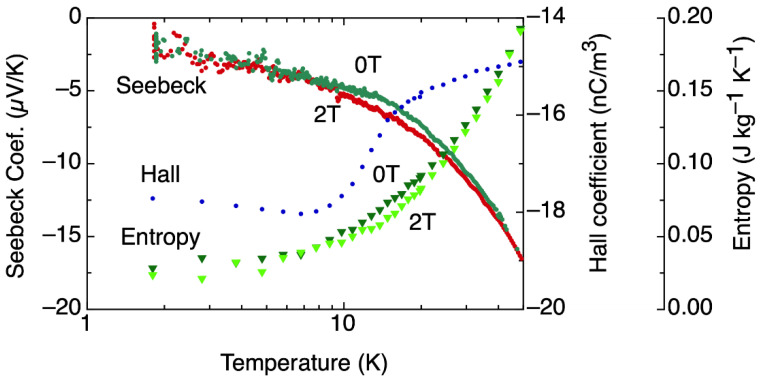
Seebeck coefficient, Hall coefficient, and entropy as a function of temperature in the vicinity of the transition region. Labels indicating the intensity of the magnetic field are shown beside the corresponding S and entropy data. The hysteresis in magnetoresistance correlates well with the additional peak in C_p_/T observed near 3 K.

The effects on the electronic structure in the transition region were also evident from our data of Hall coefficient and Seebeck coefficient, S, as shown in Figure 3. At 40 K, a small, but measurable, reduction in S is observed between zero field and 2 T, indicating changes in the electronic structure induced by the magnetic field. The Hall coefficient decreases significantly below 20 K and shows a shallow minimum at approximately 9 K, corresponding to the position of the maximum in ρ. A measure of the entropy carried by the electrons can be obtained from the Hall and S data, [26,27] and is shown in Figure 3, where a reduction in the transported entropy from zero field to 2 T is evident between 30 and 8 K. These results support the proposition that magnetic interactions result in changes in the electronic structure in clathrate-VIII Eu_8_Ga_16_Ge_30_, beginning at a temperature of approximately 40 K. Below 8 K, there is no difference in entropy of the conduction electrons as observed by S measurements with and without field.

**Figure 4:**
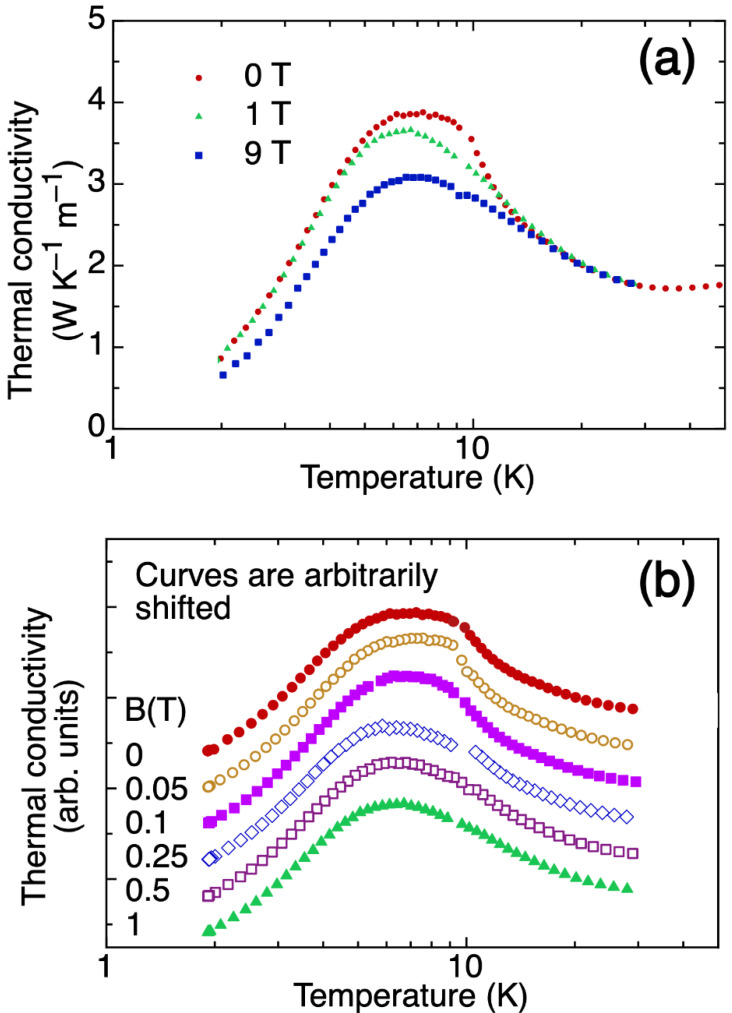
Thermal conductivity as a function of temperature for (**a**) 0 T, 1 T, and 9 T magnetic fields, and (**b**) for magnetic fields between 0 and 1 T, showing the gradual flattening of the cusp below 10 K.

Temperature- and magnetic-field-dependent κ data are shown in Figure 4. In this figure, κ = κ_L_, i.e., the lattice contribution to κ, κ_L_, dominates as the electronic contribution κ_e_ (=L_0_T/ρ, where L_0_ is the Lorenz number) is less than 1% of the total κ in the measured temperature range. Above 15 K, κ shows no field dependence, even at 9 T (Figure 4a); however, below 10 K, κ decreases with increasing field with a cusp at about 9 K that gradually flattens (see Figure 4b), following the behavior of C_p_ in the same temperature range (Figure 2d).

## 4. Conclusions

Our low temperature, electrical, and thermal measurements show that the magnetism of Eu_8_Ga_16_Ge_30_ undergoes several, gradually evolving stages from room temperature down to 2 K. Deviation from paramagnetic behavior begins well above 20 K, as can be seen by our magnetoresistance and C_p_ data. Between 20 K and 8 K, Eu_8_Ga_16_Ge_30_ retains significant traits of a glassy magnetic system where conduction electrons are involved via RKKY interaction. Energy dissipation processes occurring around 13 K, observed in AC magnetic susceptibility, result in a decrease in electronic entropy in that region when a magnetic field was applied. Below about 8 K, there is a gradual transition to what would correspond to a ferromagnet. Clear evidence for this was the hysteresis observed in the magnetoresistance, as well as an additional, albeit small, peak in C_p_ at about 4 K. It is in this temperature range that the thermal transport is most affected by the applied magnetic field. These findings suggest that phonons interact with the magnetic part of the material only when long range ferromagnetic ordering is present. Suppression of the glassy magnetic disorder, with a moderate applied magnetic field below 13 K, was revealed by the reduction in C_p_ and κ from 13 K to 4 K. Further increasing the magnetic field results in a larger reduction in C_p_ and κ down to 2 K. Our finding should motivate theoretical evaluation of the lattice dynamics of the guest–host interactions with field in this material.

## Data Availability

The data of this work is available upon reasonable request made to the authors.
